# Alleviation of secondary brain injury, posttraumatic inflammation, and brain edema formation by inhibition of factor XIIa

**DOI:** 10.1186/s12974-017-0815-8

**Published:** 2017-02-20

**Authors:** Sarah Hopp, Marc W. Nolte, Christian Stetter, Christoph Kleinschnitz, Anna-Leena Sirén, Christiane Albert-Weissenberger

**Affiliations:** 1 Department of Neurology, University Hospital Würzburg, Würzburg, Germany; 2 Department of Neurosurgery, University Hospital Würzburg, Josef-Schneider-Strasse 11, Würzburg, Germany; 3 CSL Behring GmbH, Marburg, Germany; 4 Department of Neurology, University Duisburg-Essen, Essen, Germany

**Keywords:** Focal brain lesion, Brain edema, Factor XII

## Abstract

**Background:**

Traumatic brain injury (TBI) is a devastating neurological condition and a frequent cause of permanent disability. Posttraumatic inflammation and brain edema formation, two pathological key events contributing to secondary brain injury, are mediated by the contact-kinin system. Activation of this pathway in the plasma is triggered by activated factor XII. Hence, we set out to study in detail the influence of activated factor XII on the abovementioned pathophysiological features of TBI.

**Methods:**

Using a cortical cryogenic lesion model in mice, we investigated the impact of genetic deficiency of factor XII and inhibition of activated factor XII with a single bolus injection of recombinant human albumin-fused Infestin-4 on the release of bradykinin, the brain lesion size, and contact-kinin system-dependent pathological events. We determined protein levels of bradykinin, intracellular adhesion molecule-1, CC-chemokine ligand 2, and interleukin-1β by enzyme-linked immunosorbent assays and mRNA levels of genes related to inflammation by quantitative real-time PCR. Brain lesion size was determined by tetrazolium chloride staining. Furthermore, protein levels of the tight junction protein occludin, integrity of the blood-brain barrier, and brain water content were assessed by Western blot analysis, extravasated Evans Blue dye, and the wet weight-dry weight method, respectively. Infiltration of neutrophils and microglia/activated macrophages into the injured brain lesions was quantified by immunohistological stainings.

**Results:**

We show that both genetic deficiency of factor XII and inhibition of activated factor XII in mice diminish brain injury-induced bradykinin release by the contact-kinin system and minimize brain lesion size, blood-brain barrier leakage, brain edema formation, and inflammation in our brain injury model.

**Conclusions:**

Stimulation of bradykinin release by activated factor XII probably plays a prominent role in expanding secondary brain damage by promoting brain edema formation and inflammation. Pharmacological blocking of activated factor XII could be a useful therapeutic principle in the treatment of TBI-associated pathologic processes by alleviating posttraumatic inflammation and brain edema formation.

## Background

Kinins are the key regulators of vascular permeability, edema formation, transendothelial cell migration, and inflammation after injury in different organs [1–3]. Activation of the contact-kinin system plays a detrimental role in traumatic brain injury (TBI), and its inhibition has therapeutic potential [4–10]. However, translation into clinical practice in TBI has failed thus far as it remains unclear which component of this system is best suited as a therapeutic target structure [3].

Coagulation factor XII (FXII; Hageman factor) plays a leading role in injury-induced thrombosis and inflammation [11]. Activation of FXII (generating activated FXII (FXIIa)) via contact with negatively charged surfaces or inorganic polyphosphates released from activated platelets not only triggers the intrinsic coagulation cascade but also cleaves plasma prekallikrein to form plasma kallikrein. Plasma kallikrein in turn induces the release of bradykinin from high molecular weight kininogen (for review, see [2, 11]. Plasma kallikrein can further activate FXII via a positive feedback loop [11]. As deficiency of FXII in patients is not associated with a bleeding phenotype, the contact pathway was originally thought to be dispensable for physiological hemostasis [11]. The generation of FXII knockout mice challenged this dogma as those mice exhibit severely impaired thrombus formation [12]. We recently reported that both the genetic deficiency of FXII and the pharmacologic inhibition of FXIIa prevented thrombus formation, neurodegeneration, and functional deficits after brain trauma without increasing bleeding risk, pointing to a novel treatment option [13]. Hence, we set out to study in detail the influence of FXIIa inhibition on inflammation and brain edema formation, two pathological key events that are mediated by the contact-kinin pathway after brain injuries [4, 13]. We used the cryolesion model that is particularly suited to mimic posttraumatic inflammation and brain edema formation in an extremely well-standardized and reliable fashion but lacks the contrecoup and diffuses axonal injuries that often complicate human head injuries [14]. Our results show that inhibition of FXIIa diminishes brain injury-induced bradykinin release and reduces lesion size, edema formation, blood-brain barrier damage, and inflammation.

## Methods

### Animals

A total of 243 male C57Bl/6N (wildtype) mice and 55 male FXII-deficient (FXII^−/−^) mice [15] at the age of 6 weeks were used in this study. Mice were housed in groups of three to eight with free access to food and water and a 12-h light/12-h dark cycle. In this study, all experiments were approved by institutional and regulatory authorities and were conducted in accordance with the EU Directive 2010/63/EU and the ARRIVE criteria [16].

### Cortical cryolesion model

Cortical cryolesion was induced as described previously [14]. Briefly, the mice were anesthetized with an intraperitoneal injection of ketamine (0.1 mg/g body weight) and xylazine (0.005 mg/g body weight). After restraining the mouse head in a stereotactic frame, surgery was performed on the right parietal cortex after exposing the skull through a scalp incision. A copper cylinder with a tip diameter of 2.5 mm was filled with liquid nitrogen (−196 °C) and placed on the right parietal cortex (coordinates from the bregma 1.5 mm caudal, 1.5 mm lateral) for 90 s. Sham-operated animals underwent the same surgical procedure without cooling of the copper cylinder. All surgical procedures were performed by the same person, and animals were randomly assigned to the different treatment groups.

### Pharmacological treatment

One hour after induction of focal cryolesion, a group of 74 wildtype mice received a single intravenous injection of the specific FXIIa inhibitor rHA-Infestin-4 (CSL Behring GmbH, Marburg, Germany) at a dose of 200 mg/kg body weight [13]. Control animals received equal volumes of 0.9% sodium chloride (vehicle).

### Determination of lesion size after cortical cryolesion

Mice were sacrificed 2 h, 1 day (1d), or 3 days (3d) after cryolesion; the brains were quickly removed and cut in five 1-mm-thick coronal sections using a mouse brain slice matrix (Harvard Apparatus). The slices were stained for 10 min at room temperature with 2% 2,3,5-triphenyltetrazolium chloride (TTC; Sigma-Aldrich) in 1× phosphate-buffered saline (PBS) to visualize the lesion. The lesion volume was calculated from the TTC-stained slices using the ImageJ software (Open Source, National Institutes of Health, USA).

### Determination of brain edema and blood-brain barrier leakage

Brain edema formation was calculated using the wet weight-dry weight method. Briefly, 1d after cryolesion, the brains were quickly removed and 8-mm-thick coronal sections from the injured (ipsilateral) and non-injured (contralateral) brain hemispheres were sampled. The freshly collected tissue samples were weighted to assess the wet weight. To assess the dry weight, samples were dried for 24 h at 50 °C and then weighted again. The water content (expressed as percentage) in the ipsilateral and contralateral brain hemisphere was calculated using the following formula:

$$ \left(\left(\mathrm{wet}\ \mathrm{weight}\ \hbox{--}\ \mathrm{dry}\ \mathrm{weight}\right)\ /\ \mathrm{wet}\ \mathrm{weight}\right) \times 100 $$

To determine blood-brain barrier leakage, extravasation of Evans Blue tracer into the brain parenchyma was measured fluorometrically as described previously [17]. The mice received 100 μl of 2% Evans Blue solved in 0.9% NaCl 4 h before they were sacrificed. The brains were quickly removed and 8-mm-thick coronal sections from the ipsilateral and contralateral brain hemispheres were sampled. The tissue samples were post-fixed in 4% paraformaldehyde (PFA). After fixation, the tissue samples were incubated for 24 h in 500 μl formamide at 50 °C in the dark to extract the Evans Blue dye. Then, the tissue samples were centrifuged and the fluorescence intensity of the supernatant was measured in duplicates by a fluorometer (Fluoroskan Ascent, Thermo Scientific) at an excitation wavelength of 610 nm and an emission wavelength of 680 nm. The concentration for each sample was calculated from a standard curve.

### Gene expression analysis

Real-time PCR was used to determine relative gene expression levels of genes related to inflammation in the ipsilateral cortices. Tissue homogenization, RNA isolation, and real-time PCR were performed as previously described [5]. Total RNA was prepared with a Polytron PT2100 homogenizer (Kinematica, Luzern, Switzerland) using the TRIzol Reagent (Invitrogen, Karlsruhe, Germany). Then, 250 μg of total RNA was reversely transcribed with the TaqMan Reverse Transcription Reagents (Applied Biosystems, Darmstadt, Germany) according to manufacturer’s protocol using random hexamers. Relative gene expression levels of interleukin (IL)-1β (assay ID Mm00434228_m1, Applied Biosystems), tumor necrosis factor (TNF) α (assay ID Mm00443258_m1, Applied Biosystems), CC-chemokine ligand (CCL) 2 (assay ID Mm00441242_m1, Applied Biosystems), and intracellular adhesion molecule (ICAM)-1 (assay ID Mm00516023_m1, Applied Biosystems) were quantified with the fluorescent TaqMan® technology. GAPDH and β-actin (TaqMan® Predeveloped Assay Reagents for gene expression, part numbers 4352339E and 4352341E; Applied Biosystems) were used as endogenous controls to normalize the amount of sample RNA. The real-time PCR was performed with equal amounts of cDNA in the 7500 Real-Time PCR System (Applied Biosystems) using the TaqMan® Universal 2× PCR Master Mix (Applied Biosystems). Reactions (total volume 12.5 μl) were incubated at 50 °C for 2 min, at 95 °C for 10 min followed by 40 cycles of 15 s at 95 °C, and 1 min at 60 °C. Water controls were included to ensure specificity. The 2^−ΔΔCt^ method was used for the relative quantification of gene expression [18].

### Western blot analysis

Immunoreactivity for occludin (anti-occludin, ab31721, 1:5,000, Abcam) in the lesioned cortices was detected by Western blot analysis as previously described [4]. Densitometric analysis of occludin was performed in a blinded fashion using ImageJ software with β-actin (A5441, 1:500,000, Dianova) as loading control to normalize the levels of occludin detected.

### Immunohistochemistry

Immunohistochemistry was performed as described previously [4]. The cryo-embedded mouse brains were cut into 10-μm-thick slices using a cryostat (Leica). The slices were fixed in 4% PFA in PBS for 15 min. Blocking of epitopes was achieved by pre-treatment with 5% bovine serum albumin (BSA) in PBS for 60 min to prevent unspecific binding. For the detection of macrophages/activated microglia, an anti-CD11b antibody (1:100; MCA711, AbD Serotec) and, for the detection of neutrophils, an anti-Ly-6B.2 antibody (1:100; MCA771GA, AbD Serotec) were applied. Afterwards, slides were incubated with a biotinylated anti-rat IgG (1:100; BA-4001, Vector Laboratories) in PBS containing 1% BSA overnight at 4 °C. Following the incubation with an avidin/biotin complex (Vectastain® ABC Kit, Biozol Diagnostica) for 60 min, the immune cells were visualized via addition of diaminobenzidine (Peroxidase Substrate Kit DAB SK-4100, Vector Laboratories). The slices were embedded in AquaTex (Merck). Cell numbers were counted at a 40-fold magnification in the ipsilateral hemispheres of five brain slices under a Nikon microscope Eclipse 50i equipped with the DS-U3 DS camera control unit and the NIS-Elements software (Nikon). For quantitative analysis, we used sections from near-identical brain regions for better comparison between groups. Negative controls for all immunohistological experiments included omission of either the primary or secondary antibody and gave no signals (not shown). For taking representative images, we used an Axioplan 2 Zeiss microscope equipped with a Visitron Systems Spot Insight 4 M Pixel color camera and the Spot Imaging 5.2 software (Diagnostic Instruments, Inc.).

### Enzyme-linked immunosorbent assays

For measurement of protein concentrations in plasma, whole blood was sampled in heparinized tubes. After removing the cells by centrifuging in a refrigerated centrifuge, the resulting supernatant (plasma) was used for enzyme-linked immunosorbent assays (ELISA) to measure bradykinin levels. To measure ICAM-1 levels in the plasma, a 50-fold dilution was required. To determine the levels of CCL2 and IL-1β, the ipsilateral brain hemispheres were quickly removed and homogenized (Sonopuls HD60 ultrasonic homogenizer, Bandelin, Berlin, Germany) using an extraction buffer (20 mM Tris, 250 mM sucrose, 2 mM EDTA, 10 mM EGTA, 1% TritonX-100) supplemented with complete protease inhibitor cocktail tablet (Roche Diagnostics) (1 ml/100 mg brain tissue). All ELISA were performed in duplicate according to manufacturer’s instructions (bradykinin ELISA: Bradykinin Fluorescent EIA Kit, FEK-009-01, Phoenix Pharmaceuticals; ICAM-1 ELISA: Mouse ICAM-1/CD54 Quantikine ELISA Kit, MIC100, R&D Systems; CCL2 ELISA: Mouse/Rat CCL2/JE/MCP-1 Quantikine ELISA Kit, MJE00, R&D Systems; IL-1β ELISA: Mouse IL-1 beta/IL-1F2 Quantikine ELISA Kit, MLB00C, R&D Systems). The fluorescent products for bradykinin ELISA were read at a fluorometer (Fluoroskan Ascent, Thermo Scientific) with wavelengths of 355 nm (excitation) and 460 nm (emission). All other assays were read at 450 nm at a photometer (MultiskanEX, Thermo Scientific).

### Statistics

The numbers of animals necessary to detect a standardized effect size on lesion volumes ≥0.2 on day 1 after cortical cryolesion were determined via a priori sample size calculation with the following assumptions: *α* = 0.05, *β* = 0.2, mean, and standard deviation (G*Power 3.0.10). Mice were randomly assigned to treatment groups (block randomization after cryolesion). To avoid bias, experiments were performed and analyzed in a blinded fashion.

All results were expressed as mean ± SEM. For statistical analysis, PrismGraph 5.0 software package (GraphPad Software, GraphPad Inc., La Jolla, CA, USA) was used. Data were tested for Gaussian distribution with the Kolmogorov–Smirnov test and, in the case of measuring the effects of two factors simultaneously, analyzed by two-way ANOVA with post hoc Bonferroni correction for multivariate analysis. In the case of measuring the effect of one factor, one-way ANOVA with post hoc Bonferroni correction was applied. If only two groups were compared, unpaired, two-tailed Student’s *t* test was performed. *P* values <0.05 were considered statistically significant.

## Results

### Factor XII promotes bradykinin release after focal brain injury

**Fig. 1:**
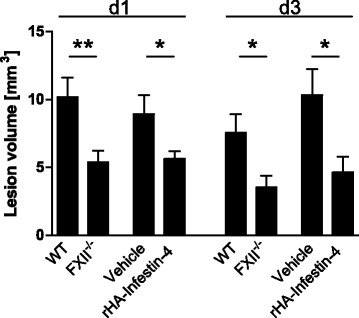
Genetic deficiency and pharmacologic inhibition of factor XIIa provides protection from tissue damage. Lesion volumetry shows less necrotic brain tissue in FXII^−/−^ mice and mice treated with rHA-Infestin-4 in comparison with control groups after 1 day (*d1*) and 3 days (*d3*), respectively (*n* = 8, **P < 0.01, *P < 0.05, unpaired, two-tailed Student’s *t* test)

Within 2 h, focal brain injury results in increased plasma bradykinin levels in wildtype and vehicle-treated mice when compared to FXII-deficient or rHA-Infestin-4-treated mice, respectively (Table 1). Accordingly, we observed diminished lesion sizes in FXII-deficient mice and rHA-Infestin-treated mice 1d and 3d after focal lesion when compared to control mice (wildtype mice and vehicle-treated mice, respectively) (Fig. 1).

**Table 1:** Determination of bradykinin plasma level by ELISA 2 h after injury induction reveals a reduction of bradykinin in FXII^−/−^ mice and animals treated with rHA-Infestin-4 similar to uninjured animals (Sham) when compared with injured wildtype (WT) and vehicle-treated mice (Vehicle) (*n* = 5 per group, **P < 0.01, *P < 0.05, one-way ANOVA followed with Bonferroni multiple comparison test and unpaired, two-tailed Student’s *t* test, respectively)

	Sham	WT	FXII^−/−^	Vehicle	rHA-Infestin-4
Bradykinin concentration (pg/ml)	2099 ± 273	6591 ± 1324	1756 ± 766*	3294 ± 683	1405 ± 266^#^

*P < 0.05 WT vs FXII^−/−^; ^#^ *P* < 0.05 Vehicle vs rHA-Infestin-4

### Brain edema formation and blood-brain barrier breakdown are dependent on factor XII

Bradykinin levels in patients with TBI correlate with the extent of edema evolution [19]. Thus, we next sought to investigate the impact of FXII on brain edema formation and blood-brain barrier function.

**Fig. 2:**
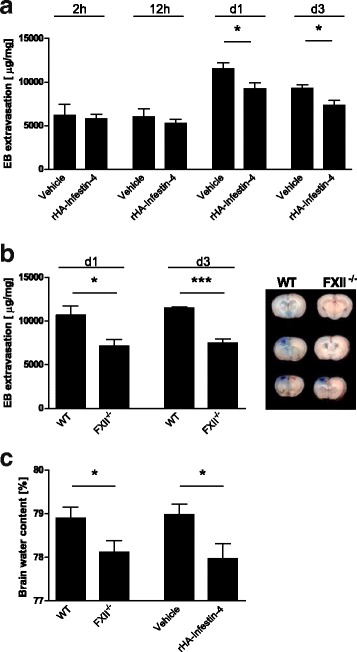
Factor XII (FXII) deficiency and pharmacologic inhibition of activated FXII displays blood-brain barrier stabilizing and antiedematous effects 1 and 3 days after induction of focal trauma. **a** Fluorometric measurement of Evans Blue extravasation into the brain parenchyma after 2 h, 12 h, 1 day (*d1*), and 3 days (*d3*) in mice treated with rHA-Infestin-4 or vehicle (*n* = 5–6 per group, *P < 0.05, unpaired, two-tailed Student’s *t* test). **b** Fluorometric measurement of Evans Blue extravasation into the brain parenchyma after 1 (*d1*) and 3 days (*d3*) in wildtype (WT) mice and FXII-deficient (FXII^−/−^) mice (*n* = 5–6 per group, ***P < 0.001, *P < 0.05, unpaired, two-tailed Student’s *t* test). *Right panel* shows representative brain slices of Evans Blue extravasation into the tissue. **c** Antiedematous effect in FXII deficiency and FXIIa inhibition shown by determination of brain water content in the lesioned hemisphere 24 h after trauma (*n* = 8–9, *P < 0.05, unpaired, two-tailed Student’s *t* test)

**Fig. 3:**
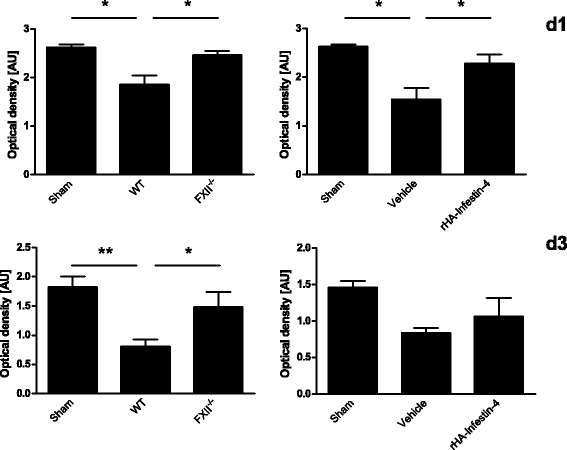
Factor XII (FXII)-deficiency and pharmacologic inhibition of activated FXII inhibits degradation of tight junction protein occludin. Quantification of occludin by Western blot analysis on day 1 (*d1*, *upper panel*) and on day 3 (*d3*, *lower panel*) after injury in sham-operated animals, WT mice and FXII^−/−^ mice (*left*), and sham-operated mice and mice treated with rHA-Infestin-4 or vehicle (*right*), respectively (*n* = 5 per group, **P < 0.01, *P < 0.05, one-way ANOVA followed by Bonferroni multiple comparison test compared with WT mice, *AU* arbitrary units)

The integrity of the blood-brain barrier, as reflected by the concentration of the vascular tracer Evans Blue leaking into the brain parenchyma, was comparable in rHA-Infestin-4-treated mice and control mice at early time points (2 and 12 h post-injury) but was preserved in rHA-Infestin-4-treated mice 1d and 3d after focal brain injury when compared to their vehicle controls (Fig. 2a). Also in FXII-deficient mice less Evans Blue leaked into the brain parenchyma 1d and 3d after focal brain injury when compared to their respective control groups (Fig. 2b). Brain edema formation as assessed by the wet weight-dry weight method was significantly reduced in FXII-deficient mice and in mice treated with rHA-Infestin-4 (Fig. 2c). We also found higher levels of the tight junction protein occludin in FXII-deficient and rHA-Infestin-4-treated mice compared to control mice (Fig. 3).

### Inflammatory processes are promoted by factor XII

**Fig. 4:**
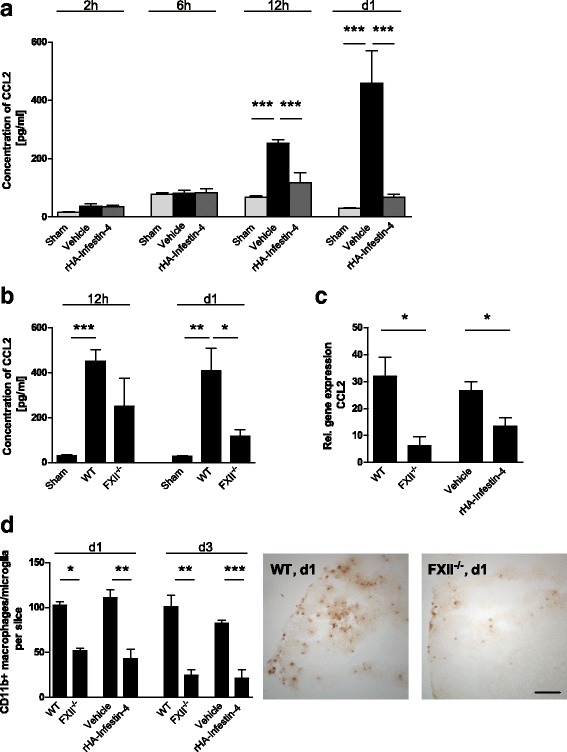
Factor XII enhances inflammatory processes after traumatic brain injury. **a** Determination of CCL2 protein concentrations in the brain tissue of sham-operated mice and mice treated with rHA-Infestin-4 or vehicle 2, 6, and 12 h, and 1 day (*d1*) after injury (*n* = 4–5 per group, ***P < 0.001, one-way ANOVA followed by Bonferroni multiple comparison test). **b** Quantification of CCL2 concentrations in the brain tissue of FXII^−/−^ mice, sham-operated mice, and wildtype controls 12 h and 1 day (*d1*) after injury (*n* = 4–5 per group, ***P < 0.001, **P < 0.01, *P < 0.05, one-way ANOVA followed by Bonferroni multiple comparison test compared with WT mice)^−/−^. **c** Relative gene expression of CCL2 in the injured cortices of FXII-deficient mice and wildtype controls, and rHA-Infestin-4- and vehicle-treated mice on day 1 after trauma induction (*n* = 5 per group, *P < 0.05, unpaired, two-tailed Student’s *t* test, amount of gene expression normalized to sham-operated animals (not shown)). **d** Macrophages and activated microglia were quantified in the lesioned hemispheres after 1 (*d1*) and 3 days (*d3*) after brain trauma (*left*). Representative immunohistochemical stainings of CD11b-positive macrophages/microglia in FXII-deficient animals and wildtype mice on *d1* (*right*). *Scale bar* depicts 100 μm (*n* = 4, ***P < 0.001, **P < 0.01, *P < 0.05, unpaired, two-tailed Student’s *t* test)

**Fig. 5:**
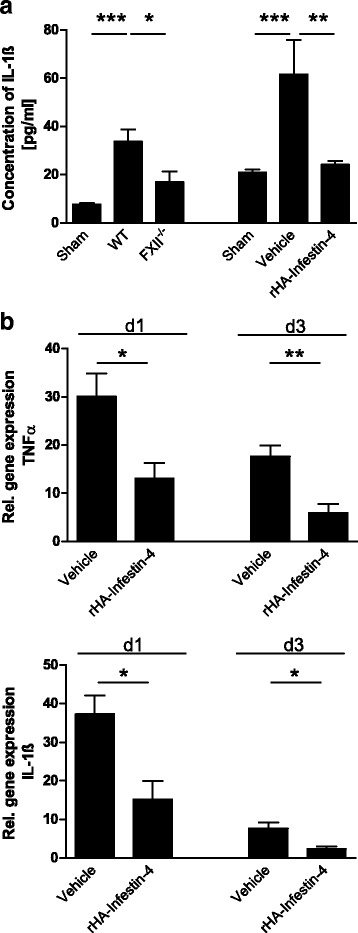
**a** Determination of interleukin-1β (IL-1β) concentrations in the brain tissue of sham-operated mice, FXII^−/−^ mice, and mice treated with rHA-Infestin-4 and their respective controls on day 1 after injury (*n* = 4–5 per group, ***P < 0.001, **P < 0.01, *P < 0.05, one-way ANOVA followed by Bonferroni multiple comparison test compared with WT mice). **b** Relative gene expression of IL-1β (*lower panel*) and tumor necrosis factor α (TNF-α, *upper panel*) in the injured cortices of rHA-Infestin-4- and vehicle-treated mice on day 1 (*d1*) and day 3 (*d3*) after trauma induction (*n* = 5 per group, **P < 0.01, *P < 0.05, unpaired, two-tailed Student’s *t* test, amount of gene expression normalized to sham-operated animals (not shown))

Bradykinin also promotes inflammatory processes, such as immune cell migration from the vasculature to sites of inflammation (for comprehensive review, see [20]). A chemokine that enhances immune cell infiltration to the site of tissue injury is CCL2 (monocyte chemoattractant protein 1, MCP-1). We therefore analyzed the time course of CCL2 tissue levels after focal brain injury. At 2 and 6 h, the levels of CCL2 in brain-injured mice remained at the same levels as in sham-treated mice. Subsequently, at 12 h and 1d, CCL2 levels were increased in vehicle-treated mice but remained on sham levels in rHA-Infestin-4-treated mice (Fig. 4a). Similarly, at 12 h and 1d, CCL2 levels were increased in wildtype mice but remained on sham levels in FXII-deficient mice (Fig. 4b). Moreover, FXII deficiency or rHA-Infestin-4 treatment resulted in significantly diminished messenger RNA (mRNA) expression of the CCL2-encoding genes when compared to the respective control groups (Fig. 4c). This was paralleled by significantly reduced migration of macrophages/activated microglia to the site of injury. More macrophages/activated microglial cells had entered the injured brain hemispheres of untreated wildtype mice when compared with mice that were deficient for FXII or were treated with rHA-Infestin-4 (Fig. 4d). Macrophages can release various inflammatory mediators after kinin stimulation including IL-1β and TNF-α (for comprehensive review, see [20]). In accordance, the protein levels of IL-1β 1d after focal brain lesions as assessed by ELISA were lower in FXII-deficient and rHA-Infestin-4-treated mice when compared to untreated wildtype and vehicle-treated mice, respectively (Fig. 5a). Significantly less mRNA of genes encoding for IL-1β and TNF-α was present in the injured cortices of rHA-Infestin-4-treated FXII-deficient mice when compared to vehicle-treated mice 1d and 3d after focal injury (Fig. 5b).

**Fig. 6:**
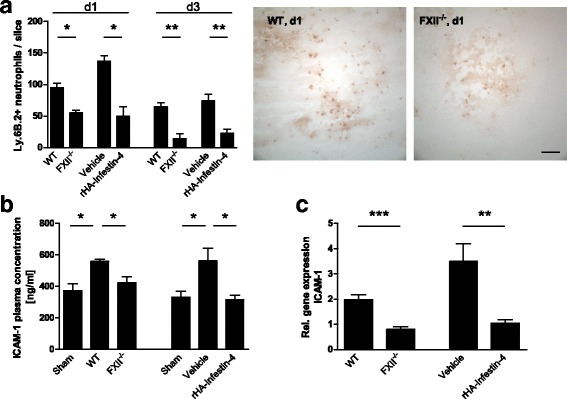
Factor XII enhances inflammatory processes after traumatic brain injury. **a** Neutrophils were quantified in the lesioned hemispheres after 1 (*d1*) and 3 days (*d3*) after brain trauma (*left*). Representative immunohistochemical stainings of neutrophils in FXII-deficient animals and wildtype mice on d1 (*right*). *Scale bar* depicts 50 μm (*n* = 4, **P < 0.01, *P < 0.05, unpaired, two-tailed Student’s *t* test). **b** Determination of ICAM-1 plasma concentrations of sham-operated mice, FXII^−/−^ mice and mice treated with rHA-Infestin-4 and their respective controls on day 3 after injury (*n* = 4–5 per group, *P < 0.05, one-way ANOVA followed by Bonferroni multiple comparison test compared with WT mice). **c** Relative gene expression of ICAM-1 in FXII-deficient mice (*left*) and rHA-Infestin-4-treated mice (*right*) in comparison to their respective control groups 3 days after trauma induction (*n* = 5, ***P < 0.001, **P < 0.01, unpaired, two-tailed Student’s *t* test, amount of gene expression normalized to sham-operated animals (not shown))

Next, we quantified the numbers of neutrophils invading the injured brain hemispheres by immunohistochemistry 1d and 3d after the induction of focal brain lesion. We observed more neutrophils in the injured brain hemispheres of untreated wildtype mice when compared to FXII-deficient mice or rHA-Infestin-4-treated mice (Fig. 6a). When we analyzed the mRNA levels of the ICAM-1-encoding genes and plasma protein levels of ICAM-1, which promote the migration of neutrophils to the site of injury [21], we found higher numbers in untreated wildtype mice when compared to FXII-deficient mice or rHA-Infestin-4-treated mice 3d after focal brain injury (Fig. 6b–c).

## Discussion

Bradykinin as the major product of the contact-kinin system triggers inflammation and brain edema formation (for a comprehensive overview, see [2]). In this study, we aimed at targeting FXIIa, the very first step required for activation of this pathway in the plasma to alleviate pathological events leading to secondary injury after brain trauma.

Following brain trauma, bradykinin levels in the cerebrospinal fluid of patients are markedly elevated up to 48 h and decrease thereafter, reaching levels of the control group within 72 h after injury [19]. Similar to the human situation, bradykinin levels maximally increase within 2 h in the brain tissue of mice after experimentally induced focal brain trauma and then subsequently decline [7]. In accordance, we observed that plasma bradykinin levels increased twofold to threefold within 2 h after focal cortical injury in mice. As FXIIa activates plasma kallikrein, it is plausible to assume that posttraumatic bradykinin release is dependent on the availability of FXIIa. Plasma bradykinin levels were significantly lower in mice deficient for FXII or treated with the specific FXIIa inhibitor rHA-Infestin-4.

Bradykinin levels in the cerebrospinal fluid of patients with brain trauma correlate with the extent of brain edema formation [19]. There is profound experimental evidence that activation of the contact-kinin system following brain trauma destabilizes the blood-brain barrier and leads to vasogenic brain edema [6–8, 22], most probably via bradykinin release [7]. Our results also point to bradykinin being critically involved in blood-brain barrier instability and brain edema formation after brain trauma. The extent of blood-brain barrier leakage and brain edema in the present study was significantly less severe in FXII-deficient mice or mice treated with rHA-Infestin-4 when compared to untreated wildtype mice.

Bradykinin is known to advance inflammation after CNS injury [23]. The inflammatory processes in turn promote posttraumatic neuronal cell loss [24–26]. In agreement with the present study, we reported earlier that blocking bradykinin receptor 1 [6] or bradykinin release by a C1 inhibitor [4] reduced invasion of macrophages and activated microglia into the damaged brain tissue and suppressed the gene expression of IL-1β and TNF-α 24 h after cortical brain injury in mice. In the current study using FXII-deficient mice or rHA-Infestin-4 treatment, we corroborated these earlier findings and showed sustained amelioration of the injury until day 3. The beneficial effects on inflammatory cell invasion were paralleled by reduced protein and mRNA levels of CCL2, a key chemokine regulating migration and infiltration of monocytes/macrophages [27] into the lesioned brain tissue. Moreover, we observed lower numbers of neutrophils and lower levels of the cell adhesion molecule ICAM-1 in FXII-deficient mice or mice treated with rHA-Infestin-4 until day 3 after focal brain lesion. Our data strongly suggest that FXIIa enhances inflammatory processes after brain trauma via activation of the contact-kinin pathway.

In addition to triggering bradykinin release, FXIIa also initiates the intrinsic coagulation pathway [11]. We showed recently that deficiency of FXII or inhibition of FXIIa improved the outcome and impeded thrombus formation in the brain microvasculature after a closed head injury in mice [13]. This beneficial effect seemed to be dependent on the activation of the intrinsic coagulation pathway as the injury-induced microvascular thrombosis, brain damage, and functional deficits could be recovered in FXII-deficient mice by the administration of hFXII but not by the administration of a recombinant hFXII variant that cannot be activated due to modifications in the activation domain [13]. However, inflammatory processes and coagulation seem to be closely interconnected (reviewed in [28–30]. As an example, von Brühl and colleagues showed that platelet-mediated leukocyte recruitment and activation contribute to the initiation and propagation of thrombosis [31]. In a mouse model of deep vein thrombosis (DVT), they revealed that platelets expressing glycoprotein Ibα contribute to thrombus formation by supporting accumulation of the innate immune cells and by binding to leukocytes [31]. Interestingly, thrombus-resident neutrophils are indispensable for subsequent DVT propagation by binding FXII and thereby supporting its activation [31]. Even if the discrete mechanisms linking inflammation and thrombosis are insufficiently investigated, it is likely that inflammation triggered by FXIIa also contributes to microvascular thrombosis.

## Conclusions

Taken together, we demonstrate here that bradykinin release is increased in our brain injury model and that this increase can be blocked by inhibition of FXIIa. Stimulation of bradykinin release seems to have a prominent role in expanding secondary brain damage by promoting brain edema formation and inflammation as all these detrimental effects can be effectively alleviated by blocking FXIIa. As inhibition of FXIIa does not increase the risk of intracranial hemorrhage [13], FXIIa is a promising target for the acute treatment of brain trauma by inhibiting two essential signaling cascades.

## Abbreviations

CCL2: CC-chemokine ligand 2
ELISA: Enzyme-linked immunosorbent assay
FXII(a): (Activated) coagulation factor XII
hFXII: Human coagulation factor XII
ICAM-1: Intercellular adhesion molecule 1
IL-1β: Interleukin-1β
PBS: Phosphate-buffered saline
PFA: Paraforamaldehyde
TBI: Traumatic brain injury
TNFα: Tumor necrosis factor α
TTC: 2,3,5-Triphenyltetrazolium chloride
